# Inferior outcome of stand-alone short versus long tibial stem in revision total knee arthroplasty. A retrospective comparative study with minimum 2 year follow-up

**DOI:** 10.1051/sicotj/2024054

**Published:** 2025-01-20

**Authors:** Elsayed Ahmed Abdelatif, Assala Abu Mukh, Ahmed Nady Saleh Elsaid, Ahmed Omar Youssef, Constant Foissey, Elvire Servien, Sebastien Lustig

**Affiliations:** 1 Department of Orthopedic Surgery and Sport Medicine, Croix-Rousse Hospital, FIFA Medical Center of Excellence 69004 Lyon France; 2 Department of Orthopaedic Surgery and Traumatology, Faculty of Medicine, Minia University 61519 Minia Egypt; 3 Orthopedics and Traumatology, Vita-Salute San Raffaele University, IRCCS San Raffaele Hospital Via Olgettina 60 20132 Milan Italy; 4 EA 7424, Interuniversity Laboratory of Human Movement Science, Université Lyon 1 69100 Lyon France; 5 Université de Lyon, Université Claude Bernard Lyon 1, IFSTTAR, LBMC UMR_T9406 69622 Lyon France

**Keywords:** Revision total knee arthroplasty, Short stem, Long stem, Pathological radiolucency

## Abstract

*Introduction:* Revision Total Knee Arthroplasty (RTKA) is complex, and induced bone loss might endanger implant fixation and joint stability. Intramedullary stems improve fixation throughout stress redistribution. The current study aims to compare the performance of short tibial stems with long tibial stems, investigating their intermediate-term radiographic and survival outcomes in RTKA. The main hypothesis is that the two types of tibial stems would exhibit similar complication and revision rates in mid-term follow-up. *Methods:* Patients who underwent RTKA for all causes in a specialized arthroplasty center from 2010 to 2022 with minimum 2-year follow-up were included in this study. Patients receiving mega prosthesis or implants associated with sleeves or cones were excluded. The final groups consisted of 234 knees: 110 patients with short stems (SS) and 124 with long stems (LS). The mean age at surgery was 65.96 ± 8.73 years in SS and 67.07 ± 8.64 years in LS. The mean Body Mass Index (BMI) was 28.95 is SS and 30.88 in LS (*p* < 0.05). The average follow-up for SS group was 4.24 years and for LS 5.16 years (*p* < 0.05). *Results:* Complications and re-revisions did not differ significantly between two groups (*p* > 0.05). Pathological radiolucency was present in 20.91% in SS group and 33.87% in LS group (*p* < 0.02). Time-to-re-revision was shorter in SS group and occurred at a mean of 3.1 years, while LS failed at a mean of 5.1 years (*p* < 0.001). *Conclusions:* The SS and LS may be comparable in terms of complications and re-revision. SS significantly fails almost 2 years earlier than long stem (*p* < 0.001). Additionally, there is a higher tendency for re-revision due to loosening in patients who present pathological radiolucency in SS group. To obtain the benefits of short stem and improve the longevity of the construct; adjuvant zone II (metaphyseal) fixation might be the clue.

## Introduction

Revision Total Knee Arthroplasty (RTKA) is continuously increasing [1] due to higher volumes of primary TKA, younger patients undergoing knee replacement, and higher demands. RTKA is complex and requires a clear identification of failure causes together with a proper surgical management.

Bone loss varies in RTKA and can challenge the surgeons’ skills [2]. Bone defects may endanger implant fixation and joint stability, reducing therefore, the construct survival [3]. The management of bone defects depends on the entity of the defect itself (its size and location). Different reconstructive options are available, such as bone cement, bone grafting, and metal augments [4]. New bone defects management options have been developed in the past years, such as metaphyseal cones and sleeves [5] that exhibit promising early results through improved fixation and reduced early loosening, however, long-term performance outcomes still lack.

Intramedullary stems were introduced to enhance fixation by bypassing bone defects and redistributing stresses throughout the bone, thereby stabilizing the revision construct [6]. Also offset stems has been developed and used in RTKA, more commonly in post-traumatic or post-osteotomy cases to accommodate the anatomical variations [7]. Stems are defined as short stems (SS) or long stems (LS), depending on whether their fixation is respectively metaphyseal or diaphyseal. Until this day, what stem fixation method to use and what is the appropriate stem length are not clearly defined [8].

To our knowledge, studies fail to directly compare the outcome of SS and LS in the revision setting in spite of their constant usage. This study directly compares the two groups evaluating their mid-term complication, radiological findings, and re-revisions. The main hypothesis was that the two types of tibial stems would exhibit similar outcome, complication and revision rates in mid-term follow-up.

## Patients and methods

### Patients

Data from patients who underwent RTKA for all causes in a specialized arthroplasty center between 2010 and 2022 were collected. The local database presented a total of 745 RTKAs. Patients who received short tibial stem or long tibial stem and who presented a follow-up longer than 2 years were included in this study. All patients receiving femoral unipolar revisions, mega prosthesis (resection implants or tumoral prosthesis), tibial components associated with cones or sleeves and patients who presented incomplete data or overall follow-up below 2 years were excluded.

**Figure 1:**
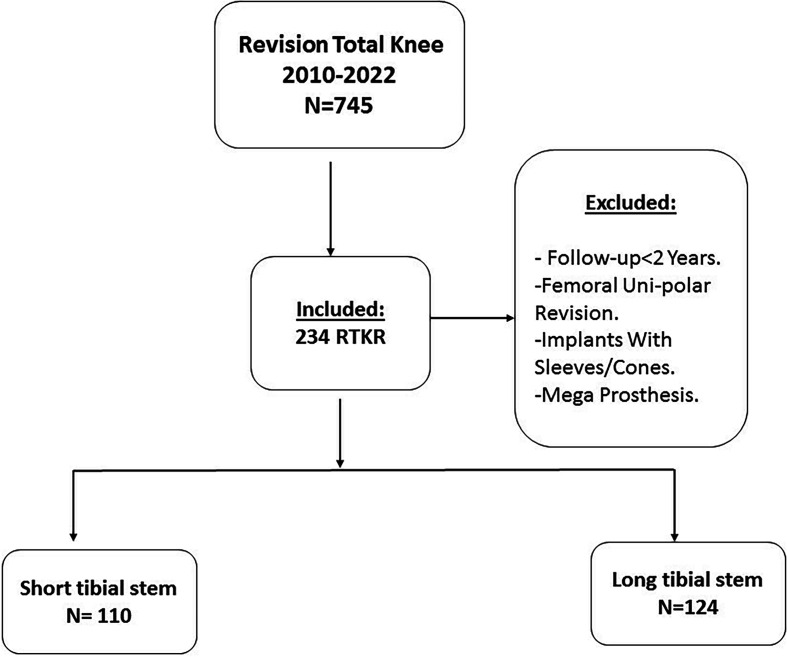
Study flowchart.

Attempts to contact patients with less than 2 years follow-up were carried; unsuccessful attempts, deceased patients, or those not meeting the inclusion criteria were excluded from this study. In the current study, we have considered a cut-off for short stems equal or less than 75 mm [8]. The final groups consisted of 110 patients who received SS (≤75 mm) and 124 patients who received LS. Figure 1 illustrates the study flowchart.

### Surgical technique

A routine pre-operative preparation was followed for all patients, all patients were operated on by experienced arthroplasty surgeons. Tourniquet was not implied, and the sub-vastus approach was utilized in the majority of cases. In case of difficult exposure, tibial tubercle osteotomy was carried out following tips and tricks of the published techniques [9]. Tissue biopsies and specimen were collected from infected or suspected infection cases for microbial examination.

Following adequate exposure, implants or antibiotic loaded cement spacer (in cases of staged revision) were removed using the combination of osteotomes and power saw. Bone loss entity was assessed and documented using the Anderson Orthopedic Research Institute (AORI) classification [10]. The choice of the stem length and offset were decided according to the case and intraoperative situation. Both stems and implant undersurface were cemented, cement with antibiotics (Palacos^®^, HERAEUS) was used in all cases except in known hypersensitive patients. Following the application of medullary cement restrictor, the cement was pressurized in a retrograde fashion using dedicated instrumentation, the real implants were fixed, and closure in layer-by-layer fashion was carried.

The same postoperative protocol was indicated for all patients unless counter-indicated. Patients were allowed for early range of motion as tolerated (except cases with tibia tubercle osteotomy where the range was kept under 90° of flexion for the first month). Patient began weight-bearing immediately with aids, and routine surgical wound care were indicated and carried out for all patients.

### Data collection

Patients were screened for demographic data; age, sex, affected side, and Body Mass Index (BMI). Pre-operative, post-operative, and additional follow-up radiographies were assessed. Radiographic assessment, data extraction and reporting were performed by two different operators who did not participate in surgeries. Clinical and instrumental records were consulted for previous implant type, cause of RTKA, bone defect entity using AORI classification, the stages of revision for infection cases, and the final RTKA implant received. The radiographic data consisted of analyzing the signs of radiolucency (RL) and were assigned to one of three groups; no RL, physiological RL, and pathological RL. Pathological radiolucency was assigned when more than 2 mm gap surrounding the implant or cement was present and progressed over time [11]. Whenever there was discordance between the investigators, a third independent operator was consulted. Outcome evaluation addressed the presence and type of complications, excluding re-revision. Analysis was carried to assess re-revisions including re-revision events, causes, and time to re-revision. Data collection respected Population, Exposure, Comparator, and Outcomes (PECO) criteria [12].

### Demographics

Patient distributions and demographics are illustrated in Table 1. There were 110 patients in the SS group and 124 in the long stem group. We observed no statistically significant difference between SS and LS regarding the mean age, sex distribution, or the affected side. The mean was 28.95 ± 4.91 in SS group, while was 30.88 ± 7.31 in the LS group. BMI was statistically significant different between the two groups (*p* < 0.05), however, this difference was not clinically significant. The mean follow-up was 4.24 years in SS group and 5.16 years in LS group, this was statistically significant (*p* < 0.05) yet, this difference was less than one year.

**Table 1:** Demographics and clinical data. SS: Short Stem, LS: Long Stem, SD: Standard Deviation, BMI: Body Mass Index, RT: Right, LT: Left, N: number, CCK: Constrained condylar knee, RHK: Rotating hinge knee, AORI classification: Anderson Orthopedic Research Institute bone defect classification.

	SS	LS	*p*-value
**Demographics**			
Number of patients	110	124	
Age (years)			*p* > 0.05
Mean ± SD (min, max)	65.96 ± 8.73 (44–85)	67.07 ± 8.64 (40–83)	
Sex			*p* > 0.05
Females	74	72	
Males	36	52	
BMI			*p* > 0.05
Mean ± SD	28.95 ± 4.91	30.88 ± 7.31	
Affected side			*p* > 0.05
RT	66	62	
LT	44	62	
Follow-up (mean in years)	4.3	5.2	*p* > 0.05
**Clinical data**			
Cause of revision			
Aseptic loosening	28 (25.45%)	31 (25%)	
Infection	28 (25.45%)	57 (45.96%)	
Instability	6 (5.45%)	11 (8.87%)	
Oversizing	13 (11.82%)	4 (3.22%)	
Stiffness	12 (10.91%)	5 (4.03%)	
Malposition	1 (0.91%)	2 (1.61%)	
PF disorder	1 (0.91%)	0	
Pain	19 (17.27%)	5 (4.03%)	
Allergy	2 (1.82%)	5 (4.03%)	
Other (Fracture)	0	4 (3.22%)	
Total	110	124	
Staged revision (infections)			
*N*	28	57	
One stage	5 (18%)	4 (7%)	
Two stages	23 (82%)	53 (93%)	
Pre-revision implant			
Primary	109 (99.01%)	83 (66.94%)	
CCK	1 (.09%)	18 (14.52%)	
RHK	–	16 (12.9%)	
Unknown	–	7 (5.65%)	
AORI			
I	103 (93.63%)	86 (69.36%)	
IIA	7 (6.37%)	31 (25%)	
IIB	–	5 (4.03%)	
III	–	2 (1.61%)	

### Ethical approval

This study received ethical approval from the Advisory Committee on Research Information Processing in the Field of Health (CCTIRS) approved this study in Paris under the protocol number 2017-A02215-48. All procedures were performed in accordance with the ethical standards of the institutional and national research committee, the 1964 Declaration of Helsinki and its later amendments, or comparable ethical standards.

### Statistical analysis

Continuous variables were described by their mean value, standard deviation and minimum and maximum values. Qualitative variables were summarized as percentages. Hypothesis analysis with student *t*-test were conducted for metric variables, *χ*^2^ tests for categorical variables, and Cox Regression for survival analysis were implied using DATAtab Team (2024) and Statistics Kingdom programs [13].

## Results

### Clinical data

Clinical data are illustrated in Table 1. Aseptic loosening and infection were the major causes of revision in the SS group, constituting almost 50% of cases while, the major cause of revision in the LS group was infection in 57 cases (46%). Primary implant (pre-revision implant) was the most frequently revised in both groups; SS group (99.1%) and LS group (66.94%). AORI I bone loss was 93.63% in the SS group and 69.36% in the LS group, this difference was not statistically significant (*p* > 0.05).

### Complications

Complications are detailed in Table 2. The most frequent complication of SS was pain (18.75%) followed by infection (14.58%) and patellofemoral complications (14.58%), while in the LS group, infection (33.33%) was the leading complication followed by fracture (19.61%). There was no statistically significant difference between the two groups regarding overall complications (*p* > 0.05).

**Table 2:** Complications, radiolucency, re-revisions. SS: Short Stem, LS: Long Stem.

	SS	LS	*p-*value
**Complications**			*p* > 0.05
Total complications	48	51	
Infection	7 (14.58%)	17 (33.33%)	
Fracture	5 (10.42%)	10 (19.61%)	
Instability	5 (10.42%)	0	
Stiffness	5 (10.42%)	6 (11.67%)	
Surgical wound disorder	0	3 (5.88%)	
Extensor mechanism failure	3 (6.25%)	4 (7.84%)	
Pain	9 (18.75%)	1 (1.96%)	
Patellofemoral complication	7 (14.58%)	2 (3.92%)	
Amputation	1 (2.08%)	1 (1.96%)	
Other (perforation, non-union, allergy, nerve palsy, patellar loosening, dislocation)	6 (12.5%)	7 (13.71%)	
**Radiolucency**			*p* < 0.02
No radiolucency	61 (55.45%)	41 (33.06%)	
Physiological	30 (27.27%)	41 (33.06%)	
Pathological	19 (17.27%)	42 (33.87%)	
**Re-revision**			*p* > 0.05
Total	15	22	
Infection	4 (26.67%)	9 (40.91%)	
Loosening	5 (33.33%)	6 (27.27%)	
Instability	2 (13.33%)	3 (13.64%)	
Fracture	1 (6.67%)	0	
Other (femoral loosening, allergy, patellofemoral complication)	2 (13.33%)	1 (4.55%)	

### Radiolucency

**Figure 2:**
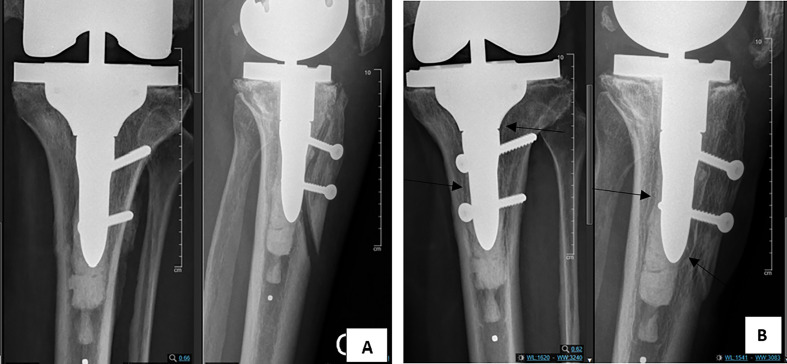
(A) Anteroposterior and lateral post operative radiographs of tibial short stem. (B) Anteroposterior and lateral radiographs of tibial short stem showing radiolucent lines (arrows) and loosening at 3 years follow-up.

**Figure 3:**
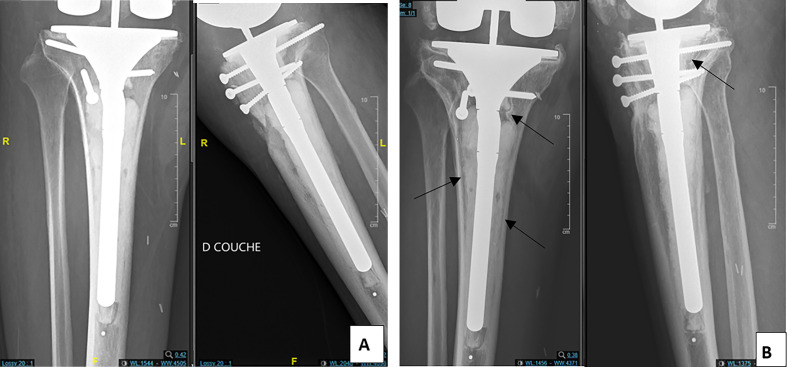
(A) Anteroposterior and lateral post operative radiographs of tibial long stem. (B) Anteroposterior and lateral radiographs of tibial long stem showing radiolucent lines (arrows) and loosening at 5 years follow-up.

Radiolucency distribution within the two groups is illustrated in Table 2. There was a statistically significant difference between the two groups regarding the occurrence of pathological radiolucency, which is higher in the LS group (*p* < 0.02), examples for pathological radiolucency are exhibited in Figures 2 and 3.

Among the cases that exhibited pathological radiolucency, a higher tendency towards re-revision for loosening was observed in SS group compared with LS group, nevertheless, the statistical difference was non-significant (*p* > 0.05).

### Re-revision

Causes of re-revision (failure) in the SS and LS groups are consultable in Table 2. Loosening was the leading cause of re-revision in the SS group, while infection caused most failures in the LS group. There was no statistically significant difference between the two groups regarding re-revision (*p* > 0.05).

**Figure 4:**
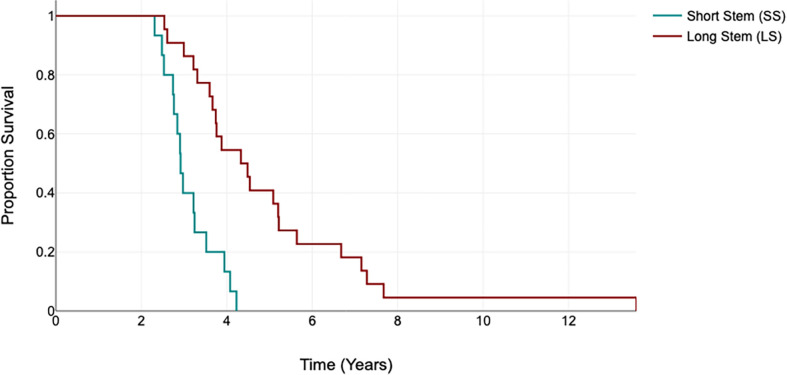
A proportional survival chart that describe time to failure in short stem and long stem groups.

Analysis was conducted to establish the time-to-re-revision. Time to re-revision in SS group occurs at a mean of 3.15 years while LS fails at a mean of 5.01 years (*p* < 0.001). The Kaplan-Meier Curve for time to re-revision are illustrated in Figure 4.

## Discussion

Despite the constant implication of stems in RTKA, the stem length and fixation method are still debatable. Each stem type has its own pros and cons, SS – that usually being cemented – allows for possible implant adjustment and antibiotic delivery through cement [14], but it may lead to more bone loss during removal of stem and cement in re-revision [15]. LS – being usually uncemented – may allow easier removal, however, it may be associated with associated with stem tip pain [14]. Regarding the method of stem fixation weather cemented or press-fit, studies failed to prove superiority of one method over the other [6, 16]. Due to the lack of direct literature comparison between SS and LS, we compare our results with available studies that investigate the performance of RTKA in study groups that included both stems, without separate group comparison, however, differences are duly addressed throughout the paper (Table 3).

**Table 3:** Literature investigate stem performance in RTKA.

Literature	Number of RTKA	Stem type	Follow-up/years	Radiolucency	Re-revision for loosening
**Stem alone**					
This study	224	110 SS	4.3	19 knees	5 knees
		124 LS	5.2	24 knees	6 knees
Lachiewicz and Soileau [17]	58	SS	5	8 knees with pathological	No
Mabry et al. [18]	70	SS	10	13 knees with variable degrees of pathological	4 knees
Whaley et al. [19]	38	16 SS	10	3 knees with pathological	1 knee
		22 LS			
Kim and Kim [20]	114	LS	7.2	3 knees with pathological	3 knees
**Stem + zone II (metaphyseal) fixation**					
Shichman et al. [21]	84 tibias	Stem + cone	2.7	No	No
Jacquet et al. [22]	66	Stem + cone	9.3	–	No
Piuzzi et al. [23]	179	Stem + cone	1.9	–	No

Complications described in literature were encountered in this study (Infections, loosening, instability, fractures, etc.) with no difference between the two groups (*p* > 0.05). It seems that our patient population presented a higher incidence of complications compared with published literatures [19, 24]. We assume this is due to our consideration of events that did not necessarily require surgical intervention (cortical perforation, surgical wound disorder, residual pain, etc.).

Our results show no statistical difference between SS and LS groups neither regarding the overall incidence of complications nor re-revisions (*p* > 0.05) (Table 2). However, we found a statistically significant difference in the incidence of pathological radiolucency, that is more frequent in the LS group (*p* < 0.02). Lachiewicz and Soileau [17] published their results with 8 out of 58 knees with pathological radiolucency (>2 mm in more than 4 zones), none of which were re-revised for loosening at the end of the follow-up. The later study seems to report both a lower incidence of pathological radiolucency and progression to re-revision for loosening, however, their population included patients with metaphyseal cones, which may enhance implant fixation and reduce loosening.

Similar to our results of pathological radiolucency incidence and its progression to re-revision due to loosening, Mabry et al. [18] reported 13 of 72 knees presented with variable degrees of pathological radiolucency and 4 knees in their series were re-revised for loosening. Worthy of notice that the latter study selected aseptic loosening patients for RTKA, and that radiographic follow-up was limited to 60% of cases at 2 or more years of follow-up.

In a study that used both SS and LS in RTKA, Whaley et al. [19] published their results after revising 38 RTKAs. Three knees showed pathological radiolucency (>2 mm), one of which was re-revised for loosening. We assume this incidence is lower than ours owing to the fact that most of their cases were primarily revised due to aseptic loosening, and half of their patients died before follow-up term.

In a study combining constrained condylar prothesis with long stems in RTKA, Kim and Kim [20] delineated that three cases were classified as pathological and subsequently revised for loosening. This incidence of pathological radiolucency is lower than our, however, all their reported cases were re-revised for loosening. Kim and Kim study primarily addresses the outcomes of a single prosthetic design without addressing the bone loss entity [20].

When addressing time to-re-revision, we observe a striking disparity between the two groups; SS group significantly fails about 2 years earlier compared with LS (*p* < 0.001). To the best of our knowledge, this literature is the sole to investigate the time to re-revision parameter. The early failure of the SS, the relatively higher re-revision for loosening in the cases with pathological radiolucency in SS group, and the higher incidence of pathological radiolucency among the LS group may raise the question: are the stand-alone stems are enough for RTKA?

Recent studies of Shichman et al. [21] and Jacquet et al. [22], investigate the results of the combination of adjuvant zone II (metaphyseal) fixation using cones and short stems, their results indicate improved stability and longevity of the revision construct. Piuzzi et al. [23] published their short-term results on RTKA using combination of metaphyseal fixation (cones) and short or long stems with 100% survivorship for aseptic loosening in SS and LS.

The performance of short stem versus long stem in RTKA may be comparable in terms of complications and re-revisions. SS fails 2 years earlier compared with LS (*p* < 0.001), additionally there is a higher tendency for re-revision due to loosening in patients who present pathological radiolucency in SS group. Despite the time-protection offered by LS against re-revisions, it shows a higher incidence of pathological radiolucency (*p* < 0.02).

### Study limitations

This study has several limitations, firstly, the retrospective and monocentric nature limits this research. In addition, the number of analyzed events might have conditioned the statistical significance of several results and very few data in literature are available to compare our results with. Lastly, even though the mean follow-up period was 4.72 years, some causes of RTKA failure occur at a greater distance of time, therefore, might have been overlooked in the current analysis

### Conclusion

Compared with LS, the use of SS in RTKA present a significant risk of early failure when used alone (around 2 years earlier). LS implication might improve implant survival, however, it is accompanied with an extensive bone removal, potential shaft pain, surgical complexity and future operative limitation. We believe that the combination of SS with zone II (metaphyseal) fixation (cones/sleeves) in RTKA might lead to both a bone preservation, and a lower incidence of aseptic loosening. This belief is also complemented with the recently published studies, however, long-term studies still needed.

## Data Availability

Data are available upon reasonable request with the corresponding author.
